# Genomic and Phytochemical Diversity Across a Collection of Snake Melon Landraces

**DOI:** 10.3390/plants14192989

**Published:** 2025-09-26

**Authors:** Maria-Dimitra Tsolakidou, Anastasia Markou, Angelos C. Kyratzis, Anastasios Kotsiras, Costas Delis, Konstadinos Mattas, Andreas Katsiotis, Nikolaos Nikoloudakis

**Affiliations:** 1 Department of Agricultural Science, Biotechnology and Food Science, Cyprus University of Technology, Limassol 3036, Cyprus; maria.tsolakidou@cut.ac.cy (M.-D.T.); ag.markou@edu.cut.ac.cy (A.M.); 2 Vegetable Crop Sector, Agricultural Research Institute—Ministry of Agriculture, Rural Development and Environment, Nicosia 1516, Cyprus; akyratzis@ari.moa.gov.cy; 3 Department of Agricultural Technology, School of Agricultural Technology and Food Technology and Nutrition, University of Peloponnese, 24100 Kalamata, Greece; a.kotsiras@go.uop.gr (A.K.); k.delis@us.uop.gr (C.D.); 4 Department of Agricultural Economics, Aristotle University of Thessaloniki, 54124 Thessaloniki, Greece; mattas@auth.gr

**Keywords:** carotenoids, chlorophyll, *Cucumis melo*, heirloom varieties, minerals, SNPs

## Abstract

Snake melons have been present for a millennia, despite their present limited use, and host a large degree of genetic and phytochemical diversity. The current study evaluated the genomic and biochemical diversity of Snake melon landraces of Cypriot and Greek origin, revealing significant degrees of genetic and mineral/phytochemical variation. Landraces showed a high potential for improving nutritional quality and a possible contribution to stress resilience. Whole-genome analysis highlighted a high degree of SNPs, InDels, SVs, and CNVs, especially in genotypes like Atzouri and ARI001024, indicating that functional variants influence phenotypic/chemical diversity. Biochemical profiling demonstrated great differences in the concentration of pigments, antioxidants, and minerals, with ARI001024 and ARI00894 exhibiting elevated levels of nutrients/phytochemicals. Hierarchical clustering and PCA analyses established relationships among traits, and reinforced the concept that these genotypes may offer valuable genetic resources for breeding programs under climate-resilient production schemes, emphasizing the need for conservation and further genomic characterization.

## 1. Introduction

Snake melon (*Cucumis melo* L. subsp. melo var. *flexuosus* L.), or Armenian cucumber, Armenian melon, yard long cucumber, faqqous, or acur, is a unique and historically significant horticultural form within the *Cucumis melo* gene pool [1]. Unlike the sweet dessert melons, Snake melon produces long, slender, non-sweet fruits—often harvested at 50–100 cm lengths—and consumed in their immature phase as cucumbers. They are relatively sweet, not bitter, with soft rinds and insipid flavor, and are appreciated raw, pickled, or as a cooked vegetable in numerous traditional cuisines.

The wild progenitors of *C. melo* are considered to be found in Africa, the Near East, India, or Central Asia [2]. In *C. melo*, domestication has apparently involved several parallel routes that led to sweet (e.g., var. cantalupensis, inodorus) and non-sweet vegetable forms (e.g., var. *flexuosus*, conomon, and chate) [3,4,5]. Genetic analysis using SSR, AFLP, SNP, and DArTseq markers indicates that var. *flexuosus* constitutes a distinct clade, representing an early split based on fruit shape (thin and long) and consumption mode (vegetable as opposed to dessert) [1,4,6,7]. The domestication of Snake melon originally took place in India and subsequently became naturalized in the Mediterranean basin, the Middle East, Asia Minor, and North Africa [8]. As a minor heirloom crop, its cultivation persists primarily in traditional, rain-fed, low-input farming systems in Lebanon, Palestine, Jordan, Syria, Egypt, Morocco, Turkey, Iran, and Spain [3]. In all these regions, it is frequently rotated with cereals or legumes and has good growth in semi-arid, saline, or poor soils, making it appropriate for farmers with fewer resources [9].

Due to its centennial cultivation, Snake melon has developed deep cultural roots. Historically, an ancestor named “faqqous” was mentioned by Pliny the Elder and medieval writers, with proto-greenhouse-like horticultural techniques rendering it available year-round [3]. In the Eastern Mediterranean, its fruit is eaten raw with lemon, salt, and yogurt; pickled; or stewed [6]. Despite its historical and cultural importance, the Snake melon is an underutilized or neglected crop. Urbanization, the presence of a series of modern hybrids in markets, and the mechanization of agriculture have reduced its cultivation. As a minor crop, it does not receive the same commercial and institutional attention that mainstream sweet melons and cucumbers get [6,9]. However, its resilience character, nutritional value, and cultural importance make Snake melon a promising crop for on-farm conservation, sustainable agriculture, and participatory plant breeding [4,9].

Minor crops like Snake melon are nowadays gaining increasing attention due to their potential contribution to biodiversity, sustainability, and low-input agriculture [10]. These underutilized crops are mostly adapted to marginal conditions and require fewer inputs, hence they are suitable for cultivation under climate change conditions and low-input farming [11]. Snake melon, in particular, is valued in Mediterranean and Middle Eastern traditional farming, where it also contributes to dietary diversification and cultural heritage [12]. Promoting the cultivation and conservation of those crops reinforces agro-biodiversity, helps resilience to pest, disease, and climatic stresses, and contributes to securing local food systems. As global agriculture seeks more sustainable and equitable models, minor crops are increasingly recognized as key components in achieving food and nutritional security [13].

However, aside from the interest in Snake melon for sustainability purposes, recent developments support a high breeding and nutritional value [14,15,16]. Such uses include anti-inflammatory, cooling, and diuretic therapies—properties now supported by laboratory verification of bioactive compounds in the seeds and leaves [17]. Comparative metabolomic and phylogenetic studies across the *C. melo* species additionally support the distinctive biochemical signature of vegetable melons. These include malic vs. sucrose/acetic acid dominance, and characteristic volatile compound profiles (moieties such as aldehydes and alcohols), highlighting unique flavor and aroma attributes [3,18,19]. Recent metabolomic profiling by mass spectrometry analysis of fruits, leaves, roots, seeds, and stems identified more than a hundred secondary metabolites in Snake melon, including flavonoids (e.g., quercetin, kaempferol), phenolic acids, cucurbitacins, lignans, and fatty acids, with leaf and seed extracts showing potent anti-inflammatory activities [3]. Nutritionally, grown fruits are also rich in K, Mg, Ca, and P and poor in Na, making them suitable for application as functional foods [9].

Since Snake melon has not been subjected to extensive breeding and genetic bottlenecks, it exhibits extremely high landrace genetic diversity, which is consistent with its open-pollinated, farmer-maintained seed systems [4]. This supports the theory that its domestication and selection have been highly decentralized and localized, promoting its adaptability to diverse ecological conditions. Indeed, seed-saved, farmer-selected landraces preserve extensive morphological and genetic diversity in terms of differences in fruit shape, ribbing, pubescence, and phenology [4,6,20,21,22]. Two main ecotypes—Baladi (lowland) and Sahouri (mountain)—were identifiable through DArTseq genotyping of over 9000 SNPs in the Levant [4]. Lebanese landraces clustered into five genetic clusters according to 10 SSR markers, revealing a plethora of distinct alleles [6]. This diversity within and between regions reflects significant evolutionary potential and enables targeted breeding, especially for stress tolerance, disease resistance, adaptability to low-input systems, and sensory quality [23]. Ongoing conservation efforts, such as Spanish Flexuosus landrace diversity mapping, in situ conservation by local farmers, and Mediterranean Genebank collections, reflect a growing recognition of its heritage and niche value. In Spain, dozens of GBS-genotyped accessions (with thousands of SNPs) expressed divergent sugar–acid profiles in immature fruits and malic acid dominance in Flexuosus/Chate groups vs. Ibericus landraces, with implications of precision breeding [9].

The goal of this study is the characterization of five Cypriot/Greek landraces of Snake melon (*Cucumis melo* var. *flexuosus*) by the combined application of genomic and biochemical methodologies. In particular, we used whole-genome genotyping to examine the structure and genetic diversity of local landraces, giving us an understanding of their genomic relationships and utility for crop improvement. Concurrently, we undertook in-depth biochemical profiling, mineral content, and major metabolic characteristics like total sugars, starch, carotenoids, and free amino acids to determine their nutritional content and functional food value. The objective of the present research is to explore the genetic and compositional diversity of these underutilized landraces, highlighting their value for conservation, nutritional value, and sustainable agriculture in semi-arid ecosystems. By the integration of genomic and metabolomic data, the study paves the way for future breeding endeavors and valorization of regional germplasm.

## 2. Materials and Methods

### 2.1. Plant Material and Experimental Design

**Figure 1:**
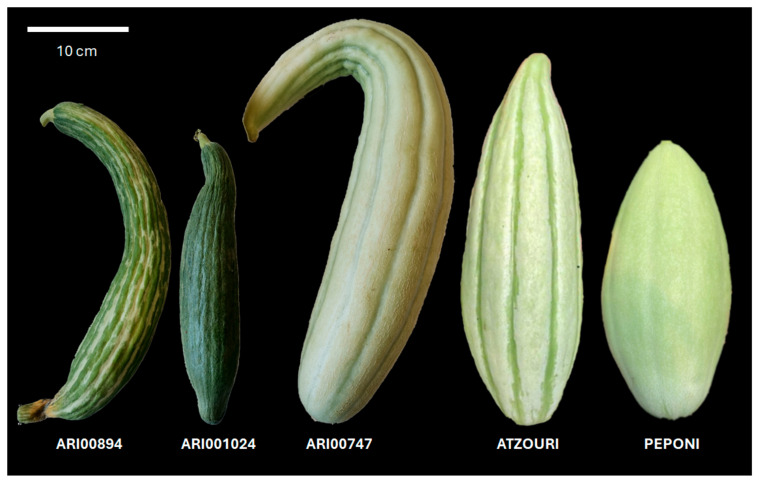
Typical fruits of the Snake melon landraces used in the current study. Phenotypical diversity across traits such as size, shape, and color is evident.

The plant material employed in this investigation consisted of five distinct landraces conserved in the Genebank of the Agricultural Research Institute (ARI), Cyprus; three Cypriot (ARI001024, ARI00894, ARI007476) and two Greek landraces (Atzouri and Peponi), as reported in Table 1. The Cypriot landraces have been registered in the national catalogue as conservation varieties. Snake melon seeds were sown in a nursery (with peat as a substrate) using a randomized complete block design with a row spacing of 50 cm and moved to a greenhouse (Fasouri, Limassol, Cyprus) at the two-leaf stage (March 2023). Three distinct blocks were used, with 10 plants per accession analyzed inside each plot. Fruit morphological features were assessed on at least 10 randomly selected fruits from the second and third trusses (Figure 1; Table 1). Drip irrigation was used every three days for 30 min. Intervals of fertilization were undertaken using an all-purpose water-soluble commercial fertilizer (20-20-20), while pest control against pests was carried out by the recurrent use of pesticides as necessary, following standard agricultural practices.

### 2.2. DNA Extraction

To extract nucleic acids, immature Snake melon leaf tips were collected and flash-frozen in liquid nitrogen. Samples were kept at −80 °C until extraction. DNA extraction was carried out using the Dneasy Plant Mini Kit (Qiagen, Hilden, Germany) according to the manufacturer’s instructions. The purity and concentration of DNA were determined using Nanodrop spectrophotometry.

### 2.3. Genomic Analyses

Whole-genome sequencing (Novogen, Newtownabbey, UK) was performed to identify genetic variations such as Single Nucleotide Polymorphisms (SNPs), insertions and deletions (InDels), Copy Number Variations (CNVs), and structural variations (SVs) in five Snake Melon landraces (Table 2, Table 3, Table 4 and Table 5). Genomic DNA was prepared and subjected to library construction using Illumina-based sequencing platform, utilizing sequencing-by-synthesis (SBS) technology. High-throughput paired-end sequencing was conducted on the Illumina HiSeq system. Quality control steps, including base calling and adapter trimming, were implemented to filter high-quality reads for further analysis. Data were analyzed to detect genetic variations, and the sequencing data for the five Snake melon landraces were aligned to the Melon_v4.0 reference genome (https://www.melonomics.net/melonomics.html#/download (accessed on 15 February 2025).

Following generation of the raw sequencing reads from the Illumina platform, data were stored in FASTQ format, containing both nucleotide sequences and their associated quality scores. A rigorous quality control process was applied to the raw data to ensure high accuracy in downstream analyses employing Fastp version 0.23.1 [24]. The distribution of base quality scores was analyzed to evaluate the sequencing accuracy. A significant percentage of the data showed quality scores of Q20 and Q30, indicating a 99% and 99.9% base calling accuracy, respectively. Low-quality reads, adapter sequences, and contaminant reads were filtered out to improve data integrity. The final clean dataset was evaluated for overall quality metrics, including total base count, GC content, and read-length distribution.

SNPs were identified by comparing the aligned reads to the reference genome using BWA. Identified SNPs were annotated based on their genomic location (e.g., exonic, intronic, or intergenic) and their potential impact on gene function. Insertions and deletions (InDels) were detected and annotated using the SAMtools suite (version 1.21) [25,26]. Structural variations (SVs), such as translocations, inversions, and duplications, were identified using a combination of read-depth and paired-end information. Structural variations (SVs) were annotated to determine their effect on gene structure and function using ANNOVAR (version 2.4) [27]. Copy Number Variations (CNVs) were detected through changes in read depth across the genome.

For the construction of the phylogenetic tree, sequence data from Meloncella chiara, SRR33529569 as reported [28], were retrieved from EBI (https://www.ebi.ac.uk/ (assessed on 20 September 2025)) and analyzed (trimmed with Fastp and aligned with BWA to the same reference genome) in order to create a VCF file (calling with bcftools). All six VCF files were normalized, harmonized, and merged into a new file that was later converted to a phylip format (vcf2phylip). The IQ-TREE software was used to produce a bootstrapped phylogenetic tree [29].

### 2.4. Minerals and Phytochemical Assessment of Fruits

Fruits (without seeds) were ground to a fine powder and filtered using a 30-mesh screen. Each sample (0.5 g) was dry-ashed in a muffle furnace at 515 °C for 5 h. The ash was then digested in 3 mL of 6 N HCl and diluted with double-distilled water to 50 mL. The contents of P (%), K (%), Ca (%), Mg (%), Na (%), Fe (mg kg^−1^), Μn (mg kg^−1^), Ζn (mg kg^−1^), Cu (mg kg^−1^), and Β (mg kg^−1^) were measured by ICP (Perkin Elmer-Optical Emission Spectrometer, OPTIMA 2100 DV, Waltham, MA, USA) as previously reported [30]. Nitrogen was measured using the Kjeldahl technique (BUCHI, digest automat K-439, and distillation Kjelflex K-360, Flawil, Switzerland). For pigments and metabolites quantification, the rainbow protocol was used [31]. Approximately 50 mg of frozen plant material (homogenized in liquid nitrogen) was subsequently treated with stepwise solvent extraction using a predetermined series of aqueous and organic solvents appropriate for each metabolite class [(*chl a* (μg/g), *chl b* (μg/g), carotenoids (μg/g), MDA (nmol/g), FAA (mg/gr), glucose (mg/g), starch, and glucose in perchloric acid (mg/g DW)]. Three biological replicates were used for the above analyses.

### 2.5. Statistical Analysis

Compositional data were subjected to an analysis of variance using R (Version 4.3.1) and RStudio suite (Version 1.2.1). A one-way ANOVA was employed, with the genotype as the main factor. The data were first tested for normality (Shapiro–Wilk test) and subsequently, post-hoc statistical Tukey’s HSD test (*p* < 0.05) was computed (Table 6). A principal components analysis (PCA) was performed to depict correlations across the phytochemical contents. The individuals were grouped by discrete color and variables by their contribution to the principal components (gradient colors). A correlation plot was also computed in order to depict positive and negative associations across the variables inquired. Finally, a normalized data array was produced, and a two-way hierarchical cluster heatmap was computed.

## 3. Results

### 3.1. Morphological Diversity

Five Snake melon genotypes were evaluated, which included three Cypriot accessions (ARI001024, ARI00894, and ARI00747) and two Greek accessions (PEPONI and ATZOURI) (Table 1). The three Cypriot genotypes differed in fruit morphology; ARI001024 produced large, dark green, slender and straight fruits, ARI00894 produced very large, green (white-striped) fruits, slender and curved, and ARI00747 produced very large cylindrical, curved and striped fruits as well. The two Greek genotypes produced large fruits of different shapes, with PEPONI producing pale green oval-shaped fruits and ATZOURI producing oblong white- and green-striped fruits. Plant growth was generally high during and after flowering in all genotypes except ARI00747, which had demonstrated medium vigor. Leaf size was large in both ARI001024 and ARI00894, and intermediate in all other accessions. Leaf senescence was moderate in all of the accessions. The sex ratio of the flowers varied with their origin. For example, the flower sex ratios for the ARI001024 and ARI00894 fruiting accessions were mainly female, however, the other study accessions demonstrated an equal amount of male and female flowers. Male flowering was late in the ARI001024 and ARI00894 genotypes, whereas it was intermediate in the other genotypes, while female flowering was early in all accessions. In all cases, an approximate width-to-length ratio ranged from 1 to 3 (PEPONI) to 1:7 (ARI00894), a typical attribute of snake melon.

### 3.2. Genomic Diversity

**Figure 2:**
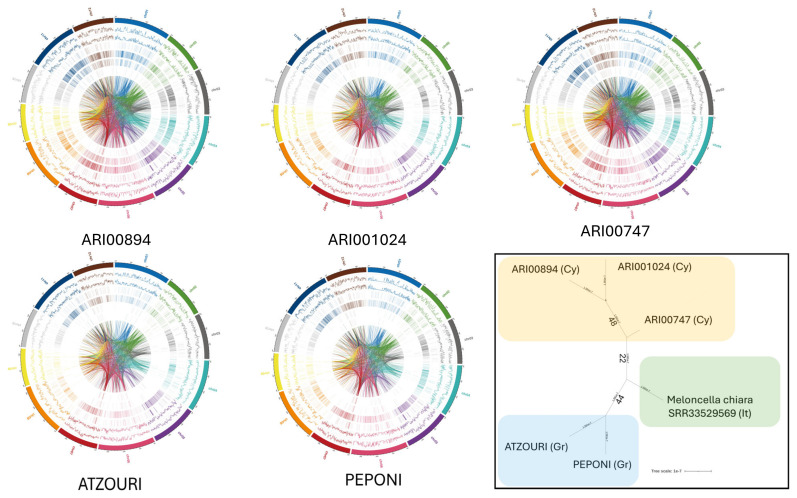
Circos plots for ATZOURI, ARI001024, ARI00894, ARI00747, and PEPONI indicated high genomic diversity within their structural and sequence features. In each plot, the chromosomes are in the outer ring, then the SNPs, InDels, CNVs duplications, CNVs deletions, SVs insertions, SVs deletions, SVs inversions, ITX, and CTX were shown completely going toward the center of the plot. Genotype SNP and InDel densities were relatively consistent across genotypes, however, regions of particular richness were observed in ARI001024 and ARI00894, suggesting polymorphic regions. CNVs duplications and deletions were more robust for ATZOURI and ARI00747, potentially suggesting more genomic expansion or contraction occurring in these genotypes. For PEPONI, structural variants, particularly insertions and deletions, were identified suggesting high levels of structural dynamics in their genome. Complex rearrangements like inversions, ITX, and CTXs were also very prominent for ARI001024 and ATZOURI, as evidenced by the many dense arcs interlinking the center of the plot. Bottom right: phylogenetic tree constructed from harmonized vcf data (SNPs) across the five Snake melon genotypes and the Meloncella chiara Italian genotype (SRR33529569) as reported [28]. A trichotomy is evident, grouping genotypes according to country of origin (Cyprus, Cy; Greece, Gr; and Italy, It).

**Figure 3:**
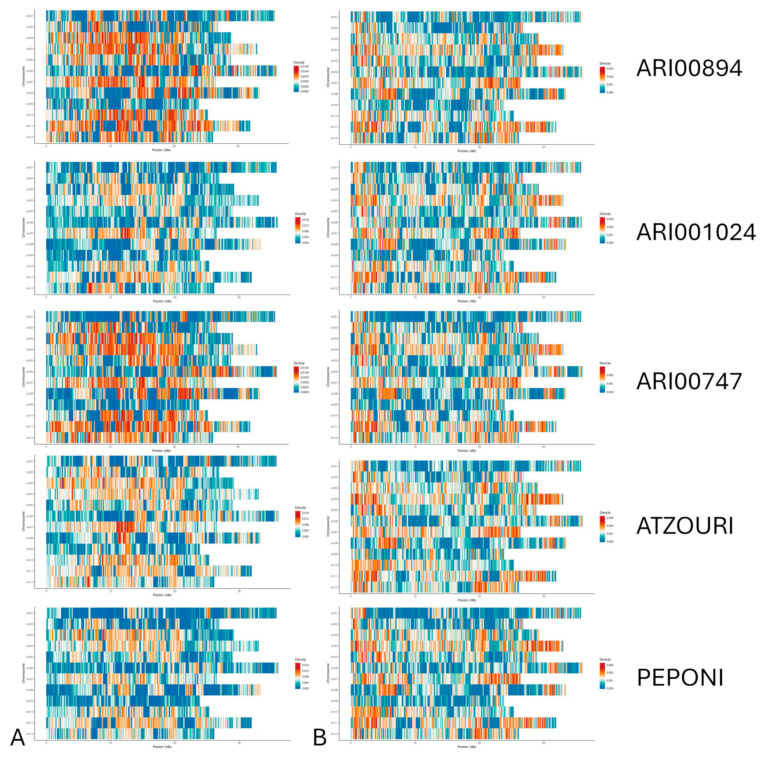
Distribution of SNPs (**A**) and InDels (**B**) across the genome of ATZOURI, ARI001024, ARI00894, ARI00747, and PEPONI landraces.

After determining sequencing quality, a detailed investigation into the genetic variants of the Snake melon accessions was conducted (Figure 2). A global characterization of the genetic variants was found, including single-nucleotide polymorphisms (SNPs) (Table 2; Figure 3), insertions and deletions (InDels) (Table 3; Figure 3), structural variations (SVs) (Table 4), and Copy Number Variations (CNVs) (Table 5). The Single Nucleotide Polymorphism (SNP) analysis among the Snake melon accessions established strong genetic variation (Table 2). Many SNPs were identified, with the highest number of SNPs in the Atzouri accession (1,946,401 total SNPs), followed by the ARI00747 (1,582,220) and Peponi (1,601,507)s accession. ARI00894 had slightly lower SNPs (1,577,822) compared to the ARI00747 and Peponi accessions. The large number of polymorphisms demonstrated considerable genetic variation among the genotypes. Atzouri contained the highest counts in both stop-gain (862) and stop-loss mutations (216), suggesting a greater number of potential functional variations that could impact agronomical traits. ARI001024 had the largest number of synonymous (27,351) and non-synonymous mutations (29,165), indicating a high potential for genetic variation while retaining function. Atzouri had the most stop-gain and stop-loss mutations at (862) stop-gain and (216) stop-loss mutations, which suggests a greater potential for functional variation. On the contrary, ARI001024 had the highest counts of synonymous (27,351) and non-synonymous mutations (29,165), indicating a high potential for genetic variation without the effect of metabolic disfunctions.

In order to delineate the functional phylogenetic relationships between the genotypes of the current study, a phylogenetic tree using biallelic SNPs was constructed. Additional Whole Genome Sequencing (WGS) data of an Italian (Salento) descent [28] were harmonized and appended to our dataset using the same reference genome. Interestingly, the bootstrapped dendrogram (Figure 2) portrayed a clear genomic cutoff based on geography, as the Cypriot genotypes were distinct from the Greek landraces and the Italian outgroup. Hence, based on the current dataset, low transregional genetic exchange was proven, highlighting the importance of regional germplasm preservation.

A substantial number of insertions and deletions (InDels) were also found, where the Atzouri landrace had the highest number of InDels (387,689), followed by Peponi (362,810), ARI001024 (361,758), ARI00894 (360,546), and ARI00747 (329,999). These markers were in alignment with the SNPs, confirming the high amount of variation within the genome of Cypriot and Greek Snake melon landraces. Moreover, Atzouri had the most frameshift deletions (1092) and insertions (985), indicating a high potential to disrupt gene function, thereby affecting phenotypic expression. ARI001024 also had a substantial number of frameshift signals with (970) deletions and (897) insertions, demonstrating a reasonable level of genetic variation. In contrast, the other genotypes had lower counts across the considered categories, although ARI00894, Peponi, and ARI00747 all had significant counts for frameshifts. Nonetheless, Atzouri and ARI001024 exhibited a generally higher incidence of disruptive mutations (Table 3).

The structural variation (SV) analysis suggested extensive variation across the Snake melon accessions. The SV analysis provided total variation counts for each sample, with Peponi having the highest total (16,503), followed by ARI001024 (15,276), ARI00894 (14,294), and Atzouri (14,290), with ARI00747 having the lowest value (13,655) (total variation represents a full range of structural variation, including deletions, insertions, duplications, and inversions). All samples appeared to mostly have deletions, with ARI001024 having 7177 deletions, whilst Atzouri demonstrated a similar amount of 6784 deletions, and Atzouri and ARI00894 had high amounts of SV in this category, which indicates the structural complexity of these genomes. Inversions were about the same as translocations for occurrence in the samples, with Peponi and ARI00894 having the largest counts of inversions (1610 and 1588, respectively) although Atzouri had the lowest number of inversions (1029). Translocations occurred in high frequencies across all accessions, where Peponi demonstrated the most counts in both intra-(2090) and inter-chromosomal translocations (6376) compared to the other accessions (Table 4).

Next, the Copy Number Variation (CNV) analysis of the Snake melon accessions showed a clear variation among the accessions. ARI001024 had a total of 6946 CNVs, which included 6,510,000 bp total duplication and 27,136,000 bp total deletion. ARI00894 had the most CNVs, at 9024. ARI00894 had 7892 deletions and 1132 duplications. Duplications totaled 6,369,400 bp, and deletions totaled 27,531,800 bp. Peponi had 8985 CNVs with 7872 deletions and 1113 duplications. The total lengths of duplications and deletions were, respectively, 6,226,800 bp and 27,889,000 bp. ARI00747 had 5296 CNVs with 4339 deletions and 957 duplications, which totaled 6,646,200 bp for duplications and 25,390,900 bp for deletions. Atzouri had 4898 CNVs with 3884 deletions and 1014 duplications, which totaled 7,693,300 bp for duplications and 29,460,900 bp for the deletions. Therefore, the data indicated extensive genomic variation among the accessions (Table 5).

### 3.3. Compositional Assessment

Profound variations among the five Snake melon landraces for their phytochemical composition and mineral nutrient content were detected (Table 6). The highest contents of *chl a* (81.5 ± 3.14 μg/g), *chl b* (43.6 ± 1.63 μg/g), and total carotenoids (24.5 ± 1.08 μg/g) were recorded for ARI001024, indicating superiority for pigment accumulation (Figure 1). ARI 00894 also recorded high chlorophyll and carotenoid content, though lower than ARI001024, in support of its position among the more biochemically superior accessions. On the contrary, the Peponi landrace recorded the lowest pigment contents, reflective of lowered biosynthesis of photosynthetic pigments.

With respect to oxidative stress and possible antioxidant capacity, malondialdehyde (MDA) content was highest in ARI00747 (12.2 ± 1.25 nmol/g), indicating greater lipid peroxidation, whereas ARI00894 and Peponi had the lowest values (8.15 ± 1.15 and 8.1 ± 0.59 nmol/g, respectively), indicating greater membrane stability. Free amino acid (FAA) content varied significantly, with ARI00894 containing the highest concentration (88.5 ± 1.48 mg/g), followed by ARI001024 and Atzouri, indicating greater protein turnover or nitrogen metabolism in these landraces.

Sugar content also varied greatly across the landraces. Soluble glucose content was highest in ARI00747 (71.2 ± 8.17 mg/g), followed by Peponi (52.8 ± 0.76 mg/g), indicating stronger sink activity and/or more active sugar metabolism in these genotypes. Interestingly, ARI001024, while having a high phytochemical profile, had relatively lower levels of soluble glucose (33.1 ± 1.27 mg/g), demonstrating that sugar is diverted to other biosynthetic pathways, such as amino acids or pigments. Glucose content after perchloric acid extraction (associated with hydrolyzed polysaccharides/starch), was more uniform across genotypes, with Atzouri (74.3 ± 5.39 mg/g DW) and ARI001024 (73.2 ± 1.84 mg/g DW) having the highest contents. This suggests that these landraces may have greater starch or complex carbohydrate reserves, which could in turn contribute to fruit texture and post-harvest quality.

Mineral nutrient analysis revealed that ARI00894 had the highest nitrogen (2.85 ± 0.06%), potassium (3.75 ± 0.35%), and iron (73.4 ± 1.67 mg/kg) content, while ARI 001024 also had a high content of N, P, Mg, and several micronutrients like Zn (22.1 ± 1.21 mg/kg) and Cu (11.3 ± 1.39 mg/kg). Peponi, however, had comparatively lower values for most of the macro and micronutrients. ARI00747 was characterized by its high glucose and intermediate levels of the remaining traits, while Atzouri had intermediate profiles but was first for Ca content (0.381 ± 0.0048%) and perchloric-acid-extracted glucose.

### 3.4. Hierarchical Clustering of Landraces, Nutrients, and Phytochemicals

**Figure 4:**
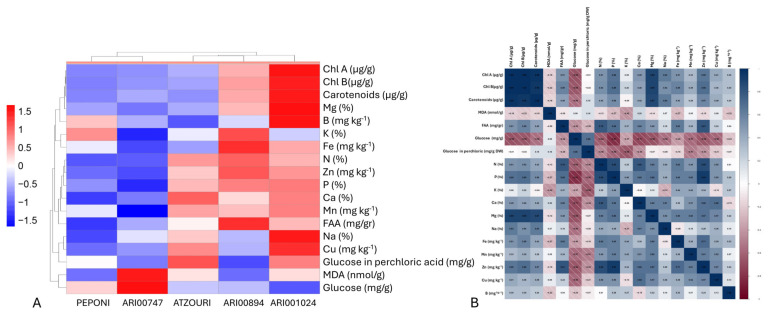
(**A**) Hierarchical heatmap clustering depicting associations among landraces and phytochemicals studied. ARI00894 and ARI001024 landraces showed an elevated concentration of nutrients. (**B**) Pearson correlations across the traits studied. Most minerals and phytochemicals have positive affiliations, except for simple and composite sugars.

The hierarchical heatmap of clustering provided an overview of the phytochemical diversity and associations among the five Snake melon landraces (Figure 4A). The clustering of the different genotypes reflected different biochemical profiles, as ARI001024 and ARI00894 were clustered with each other and together exhibited the higher levels of pigment (*chl a*, *chl b*, and carotenoids), minerals (Mg, Fe, and Cu), and glucose content, indicating a higher nutritional value than the other landraces. PEPONI and ARI00747 exhibited lower levels of these, but other traits exhibited differing elements such as free amino acid (FAA) levels and sodium (Na). The hierarchical clustering also revealed high co-associations among the chlorophylls, carotenoids, and minerals such as magnesium (Mg) and iron (Fe), which was anticipated due to their involvement in the biochemical and photosynthetic implications. Macronutrients such as nitrogen (N), phosphorus (P), and potassium (K) were also above average, indicating a coordinated transport/assimilation/movement of nutrients. Interestingly, the potential markers of stress and oxidative metabolism (malondialdehyde [MDA] and sugars (glucose)) were clustered together, possibly indicating a distinct regulated system.

To elucidate the affinity between the phytochemical content across the landraces, a correlation analysis was attempted, and a clear biochemical interdependence between phytochemical and mineral aspects of the Snake melon landraces was established (Figure 4B). The highest and most significant positive correlation was exhibited among photosynthetic pigments (*chl a*, *chl b*, and carotenoids; r > 0.96), referring to closely interrelated biosynthetic pathways. The basic minerals Mg (r > 0.87), Fe (r > 0.51), and Zn (r > 0.57) were also strongly (positively) correlated with these pigments, affirming their roles in photosynthesis and chloroplasts. Specifically, Mg exhibited an extremely high positive correlation with both chlorophylls (r > 0.85), stressing its critical role as the core atom of chlorophyll molecules. On the other hand, glucose levels were negatively correlated with a variety of key nutrients and pigments, including P (r = −0.81), Mg (r = −0.68), Ca (r = −0.71), and *chl a* (r = −0.68), suggesting trade-offs between photosynthetic or mineral activity versus sugar build-up. The clusters of macronutrients N, P, and K also showed positive correlations between them, implying coordinated acquisition or mobilization of nutrients.

**Figure 5:**
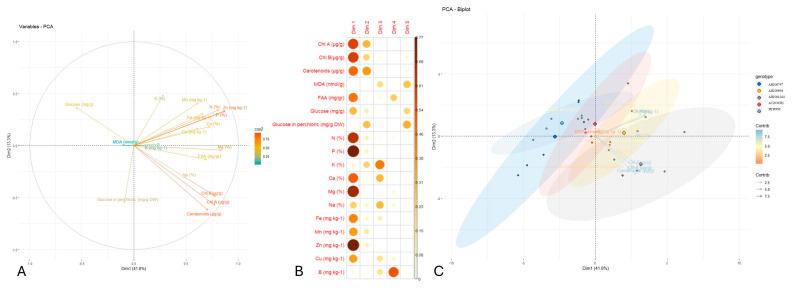
(**A**) Each trait’s (minerals/phytochemicals) contribution in the two dimensions is represented by a gradient and color intensity (scale). Vectors near the plot’s center have lower cos2 values. Narrow angles between variables suggest affinity, while wide angles show a negative association. (**B**) Quality of representation of the mineral/phytochemical variables on the factor map across dimensions. Larger size and intense color indicate a better representation. (**C**) PCA biplot across the five Snake melon landraces. Smaller circles correspond to discrete biological replicates, and larger circles match their means. Color coding corresponds to the upper-right legend.

In order to define genotypic clusters and to acknowledge the separating traits, a principal components analysis was performed (Figure 5). The PCA biplot illustrated the contribution and the interrelationships of the various variables analyzed as principal components. The first two principal components (Dim1 and Dim2) explained 41.8% and 13.3% of the total variance (approximately 50%), respectively. Most of the variables, such as carotenoids, *chl a*, *chl b*, Zn, N, and Mg, strongly aligned along the first dimension (Dim1), indicating they constitute key drivers of the first principal component. These variables also exhibited high cos^2^ values (represented in the plot by warmer colors), meaning that they were well represented in the PCA area. Strong correlations were also observed among pigments (*chl a*, *chl b*, and carotenoids), micronutrients (e.g., Zn, Fe, and Mn), and macronutrients (e.g., N, P, and Mg), suggesting coordinated regulation or mutual physiological routes. In contrast, variables like glucose, carbohydrates, and MDA were positioned closer to the origin or along different axes, implying lower contributions and/or separate roles relative to the core of the variables.

The PCA biplot indicated clear differences in genotypes with respect to their physiological and biochemical traits. The two-dimensional PCA explained 55.1% of the variance and the first principal component (PC1) explained 41.8%, demonstrating that the two-axes captured most of the variation. The genotype Peponi was in a separate cluster along the negative side of the PC1, which was closely associated with a higher MDA and sugars like glucose and sucrose, and indicated a potential stress response profile. In contrast, ARI00894 genotypes were placed positively along both axes with traits associated with Zn and K, which could imply a more favorable nutrient and growth aspect. ARI001024 and Atzouri occurred more centrally, resulting in an intermediate trait value. It is of interest to note that ARI00747 displayed more variation but was generally skewed negative to the PC1 axis, implicating a lower pigment and nutrient relationship.

## 4. Discussion

The present study carried out a systematic evaluation of the genomic and biochemical heterogeneity of Snake melon landraces that are cultivated in Cyprus and Greece, indicating substantial intraspecific variation both genetically and in the mineral/phytochemical parameters. The degree of variation is particularly remarkable as the commercial usage of Snake melon is relatively low compared to other *Cucumis melo* groups, suggesting that this genetic diversity in the landraces may be tapped to enhance nutritional quality and stress resilience or for niche and targeted breeding.

The attraction of the whole genome variant analysis is the density of the predicted SNPs, InDels, structural variants (SVs), and copy number variants (CNVs) that were distinguishable within the five landraces. Genotypes such as Atzouri and ARI001024 also had high levels of disruptive variants, including stop-gain, stop-loss, and frameshift mutations, which could signify functional variants contributing to phenotypic variation or adaptive traits. The extensive polymorphisms documented, especially the >1.9 million SNPs in Atzouri and extreme InDel frequencies, are consistent with findings in previous studies that documented considerable variation in traditional *C. melo* landraces [5,20].

The results for both SVs and CNVs further reinforced the structural complexity of these genomes. For example, Peponi had the highest number of SVs, which could imply large rearrangements of the genome, potentially impacting gene expression or metabolic regulation. High CNVs for ARI00894 and ARI001024 imply potential gene dosage effects that may contribute to phenotypic traits such as those involving metabolite accumulation or responses to environmental stress [24,32]. These results lend support to previous inferences that traditional landraces, particularly from the Eastern Mediterranean and Middle Eastern regions, hold significant unexplored genetic diversity, which has typically been shaped through hundreds of years of selection and adaptation by farmers [4,12,22].

The biochemical profiling marked significant differences in pigment, antioxidants, amino acids, sugars, and minerals among genotypes. Both ARI001024 and ARI00894 had a better phytochemical profile, with more chlorophylls and carotenoids, and the minerals iron (Fe), magnesium (Mg), and zinc (Zn). The observations corresponded with other studies, suggesting that *C. melo* var. *flexuosus* landraces are worthwhile for their pigment and micronutrient spectra, having either better photosynthetic capacity and/or nutritional value [33]. Despite Peponi being the taxon with the greatest percentage of structural variety, it was the least enriched for pigments and minerals, but showed greater soluble sugar and MDA amounts; this implies stress-induced sugar accumulation and oxidative damage [15]. The generally low sugar content of Snake melon is a characteristic of the Flexuosus group, which is also characterized by an acidic pH, in contrast to melons used as a dessert [19]. The trade-off between photosynthetic pigments and sugar accumulation was confirmed with negative correlations identifying glucose and mineral/pigment concentration. The biochemical trade-offs often represent the complexity of source–sink relationships and the order of metabolic priority in the landraces [19]. Collectively, FAA content was the highest in ARI00894, indicating high protein turnover or nitrogen assimilation in the landrace, which may be attractive to breeding programs that target both improved protein content and nitrogen use efficiency [34]. Mineral analyses also reaffirmed the nutritional advantages of ARI00894 and ARI001024, with the former containing the highest nitrogen, potassium, and iron content, indicative of their genetic resource richness as well as low markers for oxidative stress.

Additionally, there is a well-established foundation with pigment levels in plants that relates to stress responses and antioxidant activity. High chlorophyll/carotenoid amounts indicate less stressed and better environmental conditions [35,36]. The production and stability of chlorophyll is often determined by optimal conditions. Environmental stress responses to the severity of drought, salinity, or pathogen attack cause reduced levels of chlorophyll. The loss of chlorophyll is often due to the degradation of chlorophyll, or inhibited levels of chlorophyll production, which often is associated with oxidative stress levels that are significantly increased [37]. High chlorophyll tissues tend to provide greater levels of antioxidant defenses (including functionally enhanced concentrations of beneficial phytochemicals like carotenoids and phenolics) against highly reactive oxidation species in the plant’s metabolic processes that contribute to tissue cellular injury. This relationship is important in the context of plant defense, but is also important in human nutrition. Most of the (plant) foods we consume contain some chlorophyll, and dark, leafy greens (vegetables) are often recommended sources contributing to antioxidant levels and detoxification mechanisms in the human body [38,39].

The combination of hierarchical clustering, correlation, and principal components analysis (PCA) also ensured a greater understanding of the relationships among complex traits and classification of genotypes. The evident strong correlations between pigments and multiple mineral contents (specifically Mg and Fe) support findings in plant physiology that confirm these minerals’ roles in chlorophyll synthesis and photosynthetic machinery [8,40]. In addition, the PCA biplot provided an impressive separation among genotypes based on trait syndromes. Peponi presented a distance from ARI00894 and ARI001024, which clustered together with traits such as low MDA and good nutritional indicators, in terms of the same measures. The distance separation also justifies the need for integrating phenotype data with genomic analysis to fully appreciate genotype versus environment interactions and domestication history [6,14].

Overall, the structural and functional genomic diversity and associated chemotypic variation observed in this study show that these landraces are likely to be a valuable source of alleles for nutritional, stress response, and yield traits, especially in light of today’s needs for climate-resilient crops and efforts to reduce micronutrient deficiencies in human diets [41,42]. The genomic information, particularly stop-gain, frameshift, and CNV mutations, could be valuable for marker-assisted selection or genome editing that targets agronomic trait enhancement. Likewise, the higher micronutrient content and amino acids in some genotypes would be useful for biofortification and as parental lines in breeding programs that focus on food security and nutritional quality [43]. From a conservation standpoint, our results serve to emphasize the necessity for the conservation of landraces (*in situ* and *ex situ*) with distinct adaptive characteristics and a high genomic value. Further characterization undertaken through metabolomics and transcriptomics may also expose some of the regulatory networks underpinning trait expression and resilience.

## Figures and Tables

**Table 1:** Snake melon genotypes used in the current study and their morphological diversity.

Genotype	ARI00894	ARI001024	ARI00747	Atzouri	Peponi
Origin	Cypriot	Cypriot	Cypriot	Greek	Greek
Fruit shape	Slender/Curved	Slender/Straight	Cylindrical/Curved	Oblong	Oval
Fruit color	Green/White Stripes	Dark Green	White/Green Stripes	White/Green Stripes	Pale Green
Fruit size	Very large	Large	Very large	Large	Large
Plant growth (during flowering)	High	High	Medium	High	High
Plant growth (after flowering)	High	High	Medium	High	High
Leaf size	Large	Large	Intermediate	Intermediate	Intermediate
Leaf senescence	Moderate	Moderate	Moderate	Moderate	Moderate
Ratio of flowers	Mostly female	Mostly female	Equal	Equal	Equal
Earliness male	Late	Late	Intermediate	Intermediate	Intermediate
Earliness female	Early	Early	Early	Early	Early

**Table 2:** Statistics of SNP detection and annotation.

Genotype	ARI00894	ARI001024	ARI00747	Atzouri	Peponi
Upstream	104,511	108,671	102,686	128,602	106,396
Stop gain	731	745	751	862	723
Stop loss	172	191	184	216	173
Synonymous	25,805	27,351	25,391	32,553	25,644
Non-synonymous	28,023	29,165	27,698	34,108	27,573
Intronic	183,210	191,351	181,718	227,978	183,454
Splicing	452	456	391	502	406
Downstream	88,111	92,989	87,513	109,993	90,285
Upstream/Downstream	14,989	15,799	14,552	18,611	15,531
Intergenic	1,101,107	1,156,106	1,110,848	1,353,996	1,120,223
3′UTR	18,813	19,999	18,602	23,892	19,037
5′UTR	11,778	12,361	11,757	14,931	11,935
5′UTR/3′UTR	120	108	129	157	127
Others	9	12	6	12	11
ts	1,080,127	1,133,939	1,085,573	1,333,458	1,097,036
tv	497,695	521,353	496,647	612,943	504,471
ts/tv	2.17	2.175	2.186	2.176	2.175
Het rate(‰)	0.222	0.978	0.23	2.897	0.419
Total	1,577,822	1,655,292	1,582,220	1,946,401	1,601,507

Genotype: Sample name; Upstream: SNPs located within 1 kb upstream (away from transcription start site) of the gene; Non-synonymous: single nucleotide mutation with changing amino acid sequence; Stop gain/loss: nonsynonymous SNP that leads to introduction/removal of stop codon at variant site; Synonymous: single nucleotide mutation without changing amino acid sequence; Intronic: SNPs located in intronic region; Splicing: SNPs located in splicing site (within 2 bp range of intron/exon boundary); Downstream: SNPs located within 1 kb downstream (away from transcription termination site) of gene region; Upstream/Downstream: SNPs located within <2 kb of intergenic region, which is within 1 kb downstream or upstream of genes; Intergenic: SNPs located within >2 kb intergenic region; 3′UTR: variants within 3′UTR; 5′UTR: variants within 5′UTR; 5′UTR/3′UTR: variants within 3′UTR and 5′UTR, jointly; Others: SNPs located in other region; ts: transitions, point mutation that changes purine nucleotide to another purine (A ↔ G) or pyrimidine nucleotide to another pyrimidine (C ↔ T); approximately two out of three SNPs are transitions; tv: transversions, substitution of (two-ring) purine for (one-ring) pyrimidine or vice versa; ts/tv: ratio of transitions to transversions; Het rate: genome-wide heterozygous rate, calculated by ratio of heterozygous SNPs to total number of genome bases; and Total: total number of SNPs.

**Table 3:** Statistics of InDels detection and annotation.

Genotype	ARI00894	ARI001024	ARI00747	Atzouri	Peponi
Upstream	35,342	35,224	32,000	37,824	35,756
Stop gain	55	59	60	67	56
Stop loss	10	11	15	11	13
Frameshift deletion	922	970	899	1092	940
Frameshift insertion	892	897	899	985	900
Non-frameshift deletion	765	810	756	939	761
Non-frameshift insertion	777	810	764	949	779
Intronic	47,478	48,133	44,870	52,859	48,001
Splicing	173	169	156	197	164
Downstream	27,941	28,136	25,498	30,133	28,226
Upstream/Downstream	5068	5105	4638	5533	5241
Intergenic	229,914	229,939	208,755	244,391	230,669
3′UTR	6626	6835	6317	7560	6698
5′UTR	4574	4662	4368	5162	4603
5′UTR/3′UTR	50	40	43	54	51
Others	0	0	0	0	0
Insertion	183,594	183,926	167,990	197,417	185,568
Deletion	176,902	177,659	161,956	189,828	177,121
Het rate(‰)	0.096	0.201	0.073	0.503	0.134
Total	360,546	361,758	329,999	387,689	362,810

Genotype: Sample names; Upstream: InDels located within 1 kb upstream (away from transcription start site) of gene; Stop gain/loss: InDel that leads to introduction/removal of stop codon at variant site; Frameshift deletion/insertion: InDel mutation changing open reading frame with deletion or insertion; Non-Frameshift deletion/insertion: InDel mutation without changing open reading frame with deletion or insertion sequences of 3 or multiple of 3 bases; Intronic: InDel located in intronic region; Splicing: InDel located in splicing site (2 bp range of intron/exon boundary); Downstream: InDel located within 1 kb downstream (away from transcription termination site) of gene region; Upstream/Downstream: InDel SNPs located within <2 kb of intergenic region, which is 1 kb downstream or upstream of the genes; Intergenic: InDel located within >2 kb intergenic region; 3′UTR: variants within 3′UTR; 5′UTR: variants within 5′UTR; 5′UTR/3′UTR: variants within 5′UTR and 3′UTR, jointly; Others: InDel located in other region; Het rate: InDel heterozygous rate, calculated by ratio of InDels to total number of genome bases; and Total: total number of InDels.

**Table 4:** Statistics of SV detection and annotation.

Genotype	ARI00894	ARI001024	ARI00747	Atzouri	Peponi
Upstream	458	566	484	515	431
Exonic	2685	2341	2120	2045	2702
Downstream	344	400	318	381	353
Intronic	340	401	353	397	361
Upstream/Downstream	64	90	69	77	78
Intergenic	3839	4546	4020	4344	4023
Splicing	4	5	2	7	5
Others	75	88	85	82	84
INS	51	1	0	35	98
DEL	6170	7177	6348	6784	6329
INV	1588	1259	1103	1029	1610
ITX	2010	1479	1623	1414	2090
CTX	4475	5360	4581	5028	6376
Total	14,294	15,276	13,655	14,290	16,503

Genotype: Sample names; Upstream: SVs located within 1 kb upstream (away from transcription start site) of gene; Exonic: SVs located in exonic region; Intronic: SVs located in intronic region; Downstream: SVs located within 1 kb downstream (away from transcription termination site) of gene region; Upstream/Downstream: SVs located within <2 kb of intergenic region, which is within 1 kb downstream or upstream of the genes; Intergenic: SVs located within >2 kb intergenic region; Splicing: SVs located in splicing site (2 bp range of intron/exon boundary); Others: SVs located in other region; INS: Insersion; DEL: Deletion; INV: Inversion; ITX: intra-chromosomal translocations; CTX: inter-chromosomal translocations; and Total: total number of SVs.

**Table 5:** Statistics of CNV detection and annotation.

Genotype	ARI00894	ARI001024	ARI00747	Atzouri	Peponi
Upstream	399	337	257	199	425
Exonic	1576	1272	1077	1184	1466
Intronic	289	206	133	108	301
Downstream	350	272	196	174	366
Upstream/Downstream	42	40	34	24	48
Intergenic	6278	4749	3546	3161	6283
Others	90	70	53	48	96
Duplication	1132	1063	957	1014	1113
Deletion	7892	5883	4339	3884	7872
Duplication length (bp)	6,369,400	6,510,000	6,646,200	7,693,300	6,226,800
Deletion length (bp)	27,531,800	27,136,000	25,390,900	29,460,900	27,889,000
Total	9024	6946	5296	4898	8985

Genotype: Sample names; Upstream: CNVs located within 1 kb upstream (away from transcription start site) of gene; Exonic: CNVs located in exonic region; Intronic: CNVs located in intronic region; Downstream: CNVs located within 1 kb downstream (away from transcription termination site) of gene region; Upstream/Downstream: CNVs located within <2 kb of intergenic region, which is within 1 kb downstream or upstream of genes; Intergenic: CNVs located within >2 kb intergenic region; Others: CNVs located in other region; Duplication: CNVs with increased copy number; Deletion: CNVs with decreased copy number; Duplication length (bp): total length of CNV duplication; Deletion length (bp): total length of CNV deletion; and Total: total number of CNVs.

**Table 6:** Snake melon phytochemicals across the five landraces.

	ARI00894	ARI001024	ARI00747	Atzouri	Peponi
*chl a* (μg/g)	51.3 ± 1.09 ^b^	81.5 ± 3.14 ^a^	14.2 ± 0.601 ^cd^	20.4 ± 2.37 ^c^	11.8 ± 0.227 ^d^
*chl b* (μg/g)	30.9 ± 0.718 ^b^	43.6 ± 1.63 ^a^	11.5 ± 2.3 ^c^	12.8 ± 2.69 ^c^	9 ± 0.833 ^c^
Carotenoids (μg/g)	16.1 ± 0.621 ^b^	24.5 ± 1.08 ^a^	6.43 ± 0.845 ^c^	4.85 ± 0.591 ^c^	4.28 ± 0.298 ^c^
MDA (nmol/g)	8.15 ± 1.15 ^b^	10.1 ± 0.228 ^ab^	12.2 ± 1.25 ^a^	10.1 ± 0.694 ^ab^	8.1 ± 0.589 ^b^
FAA (mg/gr)	88.5 ± 1.48 ^a^	73.9 ± 3.75 ^b^	57.8 ± 2.33 ^c^	68 ± 1.81 ^b^	46.9 ± 1.91 ^d^
Glucose (mg/g)	43.1 ± 2.44 ^bc^	33.1 ± 1.27 ^c^	71.2 ± 8.17 ^a^	43.9 ± 3.07 ^bc^	52.8 ± 0.762 ^b^
Glucose in perchloric acid (mg/g DW)	65.7 ± 3.05	73.2 ± 1.84	66.8 ± 6.82	74.3 ± 5.39	68.9 ± 1.49
N (%)	2.85 ± 0.0613 ^a^	2.62 ± 0.134 ^a^	1.97 ± 0.117 ^b^	2.69 ± 0.107 ^a^	1.95 ± 0.0636 ^b^
P (%)	0.493 ± 0.0117 ^a^	0.494 ± 0.00413 ^a^	0.37 ± 0.0157 ^b^	0.472 ± 0.00649 ^a^	0.406 ± 0.00592 ^b^
K (%)	3.75 ± 0.353 ^a^	2.98 ± 0.0205 ^bc^	2.41 ± 0.114 ^c^	3.1 ± 0.121 ^ac^	3.54 ± 0.125 ^ab^
Ca (%)	0.351 ± 0.0135 ^a^	0.374 ± 0.0106 ^a^	0.307 ± 0.00611 ^b^	0.381 ± 0.00481 ^a^	0.291 ± 0.00567 ^b^
Mg (%)	0.241 ± 0.00886 ^b^	0.279 ± 0.0079 ^a^	0.193 ± 0.00201 ^c^	0.206 ± 0.00641 ^c^	0.204 ± 0.00517 ^c^
Na (%)	0.094 ± 0.00204 ^bc^	0.138 ± 0.0126 ^a^	0.1 ± 0.0043 ^bc^	0.112 ± 0.00521 ^ab^	0.077 ± 0.00227 ^c^
Fe (mg kg^−1^)	73.4 ± 1.67 ^a^	67.2 ± 3.29 ^ab^	53.7 ± 1.76 ^c^	58.1 ± 3.82 ^bc^	61.9 ± 2.36 ^bc^
Μn (mg kg^−1^)	9.01 ± 0.273 ^ab^	10 ± 1.1 ^a^	3.9 ± 0.216 ^b^	9.5 ± 1.29 ^a^	7.65 ± 2.46 ^ab^
Ζn (mg kg^−1^)	24.4 ± 0.878 ^a^	22.1 ± 1.21 ^a^	11.3 ± 0.992 ^b^	20.2 ± 1.27 ^a^	12.5 ± 0.929 ^b^
Cu (mg kg^−1^)	7.03 ± 0.812 ^bc^	11.3 ± 1.39 ^a^	6.52 ± 0.633 ^bc^	9.93 ± 1.13 ^ab^	5.95 ± 0.358 ^c^
Β (mg kg^−1^)	18.4 ± 2.96	22.2 ± 1.92	17.8 ± 0.403	16.6 ± 0.836	19.8 ± 1.24

Values are means ± SEMs, n = 6 per treatment group, and correspond to dry weight. Means in a row without a common superscript letter differ (*p* < 0.05) as analyzed by one-way ANOVA and the TUKEY test.

## Data Availability

The raw genomic data have been deposited to NCBI SubmissionID: SUB15551345, BioProject ID: PRJNA1309765; BioSample accessions: SAMN50752992, SAMN50752993, SAMN50752994, MN50752995, and SAMN50752996; http://www.ncbi.nlm.nih.gov/bioproject/1309765 (accessed on 15 February 2025).
